# Second monoclinic form of (*E*)-3-(4-fluoro­phen­yl)-1-phenyl­prop-2-en-1-one

**DOI:** 10.1107/S1600536813028079

**Published:** 2013-10-23

**Authors:** Saira N. Arias-Ruiz, Nancy Romero, Carlos E. Lobato-García, Abraham Gómez-Rivera, Angel Mendoza

**Affiliations:** a División Académica de Ciencias Básicas, Universidad Juárez Autónoma de Tabasco, AP 24, 86690 Cunduacán, Tab., Mexico; b Centro de Química, Instituto de Ciencias, Benemérita Universidad Autónoma de Puebla, 72570 Puebla, Pue., Mexico

## Abstract

The unit-cell dimensions and space group of the second monoclinic polymorph of the title compound, C_15_H_11_FO, differ from those of the previously reported form [Jing (2009 ▶). *Acta Cryst.* E**65**, o2515]. The title compound shows an *E* conformation of the C=C bond with the 4-fluoro­phenyl group opposite to the benzoyl group. The torsion angle of between the planes of the 4-fluoro­phenyl and benzoyl groups is 10.53 (6)°. In the crystal, weak C—H⋯O and C—H⋯F inter­actions form a cross-linked packing motif, building sheets parallel to (-102).

## Related literature

For the first monoclinic polymorph of the title compound, see: Jing (2009 ▶). For related crystal structures, see: Li *et al.* (1992 ▶); Li & Su (1994 ▶); For biological properties reports of chalcones, see: Foresti *et al.* (2005 ▶); Nowakowska (2007 ▶); Kouskoura *et al.* (2008 ▶); Zhang *et al.* (2010 ▶); Doan & Tran (2011 ▶). For solvent-free synthesis of chalcones, see: Srivastava (2008 ▶); Krishnakumar & Swaminathan (2011 ▶); Thirunarayanan *et al.* (2012 ▶). For applications of chalcones in organic synthesis, see: Prakash *et al.* (2009 ▶); Bandgar *et al.* (2009 ▶).
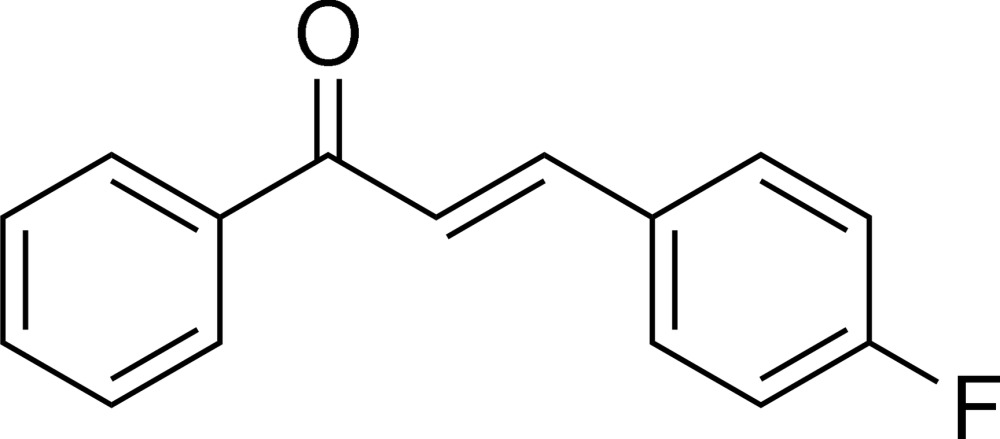


## Experimental

### 

#### Crystal data

C_15_H_11_FO
*M* *_r_* = 226.24
Monoclinic, 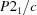

*a* = 8.6925 (4) Å
*b* = 5.9266 (2) Å
*c* = 22.6456 (9) Å
β = 95.423 (4)°
*V* = 1161.41 (8) Å^3^
*Z* = 4
Mo *K*α radiation
μ = 0.09 mm^−1^
*T* = 293 K
0.59 × 0.15 × 0.07 mm

#### Data collection

Oxford Diffraction Xcalibur (Atlas, Gemini) diffractometer
Absorption correction: analytical (*CrysAlis PRO*; Oxford Diffraction, 2009 ▶) *T*_min_ = 0.993, *T*_max_ = 0.999
22101 measured reflections
2276 independent reflections
1471 reflections with *I* > 2σ(*I*)
*R*_int_ = 0.044

#### Refinement

*R*[*F* ^2^ > 2σ(*F* ^2^)] = 0.045
*wR*(*F* ^2^) = 0.131
*S* = 1.01
2276 reflections
155 parameters
H-atom parameters constrained
Δρ_max_ = 0.11 e Å^−3^
Δρ_min_ = −0.11 e Å^−3^

### 

Data collection: *CrysAlis PRO* (Oxford Diffraction, 2009 ▶); cell refinement: *CrysAlis PRO*; data reduction: *CrysAlis PRO*; program(s) used to solve structure: *SHELXS2013* (Sheldrick, 2008 ▶); program(s) used to refine structure: *SHELXL2013* (Sheldrick, 2008 ▶); molecular graphics: *ORTEP-3 for Windows* (Farrugia, 2012 ▶); software used to prepare material for publication: *WinGX* (Farrugia, 2012 ▶).

## Supplementary Material

Crystal structure: contains datablock(s) global, I. DOI: 10.1107/S1600536813028079/bh2484sup1.cif

Structure factors: contains datablock(s) I. DOI: 10.1107/S1600536813028079/bh2484Isup2.hkl

Supplementary material file. DOI: 10.1107/S1600536813028079/bh2484Isup3.cml

Additional supplementary materials:  crystallographic information; 3D view; checkCIF report

## Figures and Tables

**Table 1:** Hydrogen-bond geometry (Å, °)

*D*—H⋯*A*	*D*—H	H⋯*A*	*D*⋯*A*	*D*—H⋯*A*
C5—H5⋯O1^i^	0.93	2.49	3.244 (2)	138
C13—H13⋯F1^ii^	0.93	2.68	3.465 (2)	142

Symmetry codes: (i) 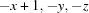; (ii) 
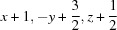
.

**Fig. 1.**
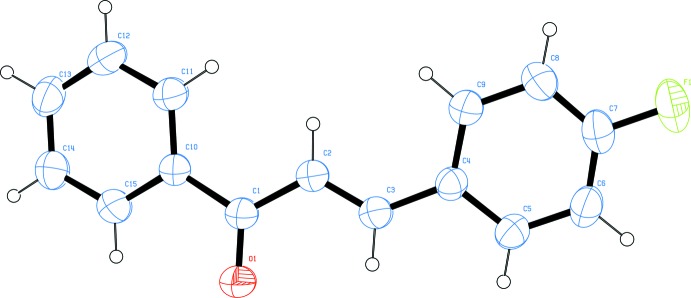
The molecular structure of title compound, with 30% probability displacement ellipsoids for non-H atoms.

## supplementary crystallographic information

### 1. Comment

Chalcones, like the title compound, are highly interesting materials due to their antioxidant, antibacterial, antifungal, antitumor and anti-inflammatory properties (Zhang *et al.*, 2010; Kouskoura *et al.*, 2008; Nowakowska, 2007; Foresti *et al.*, 2005 and Doan & Tran, 2011). The title compound was obtained by a green Claisen-Schimdt condensation of 4-fluorobenzaldehyde with acetophenone. This reaction was carried out by a free solvent and microwave assisted method, with *p*-toluenesulfonic acid as catalyst (Thirunarayanan *et al.*, 2012; Krishnakumar & Swaminathan, 2011; Srivastava, 2008). Also, chalcones are valuable intermediates in organic synthesis because of the α,β-unsaturated carbonyl system, which is considered as a key building block (Prakash *et al.*, 2009; Bandgar *et al.*, 2009).

The crystal structure of the title compound, C_15_H_11_FO, has been reported previously at 93 K (Jing, 2009). However, here we report the structure of a second monoclinic polymorph, which was elucidated at 293 (2) K. The new polymorph crystallizes in space group *P*2_1_/*c*, which is different from the previous space group, *Cc*. The cell parameters of the current monoclinic polymorph vary significantly from the earlier form [*a* = 24.926 (9), *b* = 5.6940 (19), *c* = 7.749 (3) Å and *β* = 94.747 (5)°]. Furthermore, two more halo-chalcones were reported previously: 4-bromochalcone [Li *et al.*, 1992; unit-cell parameters: *a* = 29.027 (7), *b* = 7.26 (2), *c* = 5.917 (3) Å and *β* = 101.38 (3)°] and 4-chlorochalcone [Li & Su, 1994; unit-cell parameters: *a* = 8.211 (2), *b* = 5.869 (2), *c* = 25.291 (5) Å and *β* = 99.18 °]. Both compounds show similar cell parameters to current polymorphs of the F-chalcone, however the space group is *P*2_1_/*c* for the Cl derivative and *Cc* for the Br-chalcone, both characterized at room temperature.

The title compound shows a configuration *E* on the C═C bond with *p*-fluorophenyl group opposite to the 1-phenylketone. Torsion angle of *p*-fluorophenyl to 1-phenylketone group is 10.53 (6)°. The crystal packing presents two intermolecular interactions of the type hydrogen bond (Table 1), C5—H5···O1 and C13—H13···F1 with the symmetry codes (i) -*x* + 1, -*y*, -*z* and (ii) *x* + 1, -*y* + 3/2, *z* + 1/2, respectively. These interactions form a cross-linked crystal packing, building sheets parallel to the (102) plane.

### 2. Experimental

To a mixture of acetophenone (1.15 mmol) and 4-fluorobenzaldehyde (1.15 mmol) in dry media, 0.1 g of *p*-TsOH was added and the mixture was irradiated in a microwave oven at 480 W for 10 min. Completion of the reaction was tested by thin layer chromatography (TLC), and the crude product was purified by column chromatography on silica gel (eluent: hexane), to afford a yellow solid, (*E*)-3-(4-fluorophenyl)-1-phenylprop-2-en-1-one, in 85.4% yield, m. p. 79 °C. The pure product was recrystallized from hexane. The microwave oven VICHI: MW-600 (600 W) MOD: MICV/Vichy/F-814 was used; all the chemicals were purchased from Sigma-Aldrich and were used without further preparation. Spectroscopic analysis: *ν*_max_/cm^-1^ (neat KBr)= 3438, 1661, 1604, 1588, 1509, 1217, 1016, 829, 772. ^1^H-NMR (400 MHz, CDCl_3_): δ (p.p.m.) = 7.09 (tt, *J*= 2.0, 8.4 Hz, 2H), 7.46 (d, *J*=16 Hz, 1H), 7.49 (dd, *J*= 6.4, 8.4 Hz, 2H), 7.56 (dt, *J*=1.2, 6.4 Hz, 1H), 7.61 (ddt, *J*=2.0, 5.2, 8.4 Hz, 2H), 7.76 (d, *J*=16 Hz, 1H), 8.01 (dd, *J*=1.2, 8.4 Hz, 2H). ^13^C-NMR (101 MHz, CDCl_3_): δ (p.p.m.) = 115.91, 116.13, 121.58, 128.38 (2 C), 128.56 (2 C), 130.22, 130.31, 132.77, 137.98, 143.38, 162.69, 165.19, 190.15.

### 3. Refinement

H atoms linked to C atoms were placed in idealized positions and refined as riding on their parent atoms, with C—H = 0.93 Å and with *U*_iso_(H) = 1.2 *U*_eq_(carrier C).

### Crystal data

**Table:**

C_15_H_11_FO	*F*(000) = 472
*M**_r_* = 226.24	*D*_x_ = 1.294 Mg m^−^^3^
Monoclinic, *P*2_1_/*c*	Melting point: 352 K
Hall symbol: -P 2ybc	Mo *K*α radiation, λ = 0.71073 Å
*a* = 8.6925 (4) Å	Cell parameters from 4193 reflections
*b* = 5.9266 (2) Å	θ = 3.6–23.2°
*c* = 22.6456 (9) Å	µ = 0.09 mm^−^^1^
β = 95.423 (4)°	*T* = 293 K
*V* = 1161.41 (8) Å^3^	Prism, colourless
*Z* = 4	0.59 × 0.15 × 0.07 mm

### Data collection

**Table:**

Oxford Diffraction Xcalibur (Atlas, Gemini) diffractometer	2276 independent reflections
Graphite monochromator	1471 reflections with *I* > 2σ(*I*)
Detector resolution: 10.5564 pixels mm^-1^	*R*_int_ = 0.044
ω scans	θ_max_ = 26.1°, θ_min_ = 2.8°
Absorption correction: analytical (*CrysAlis PRO*; Oxford Diffraction, 2009)	*h* = −10→10
*T*_min_ = 0.993, *T*_max_ = 0.999	*k* = −7→7
22101 measured reflections	*l* = −27→27

### Refinement

**Table:**

Refinement on *F*^2^	Secondary atom site location: difference Fourier map
Least-squares matrix: full	Hydrogen site location: inferred from neighbouring sites
*R*[*F*^2^ > 2σ(*F*^2^)] = 0.045	H-atom parameters constrained
*wR*(*F*^2^) = 0.131	*w* = 1/[σ^2^(*F*_o_^2^) + (0.0624*P*)^2^ + 0.1205*P*] where *P* = (*F*_o_^2^ + 2*F*_c_^2^)/3
*S* = 1.01	(Δ/σ)_max_ < 0.001
2276 reflections	Δρ_max_ = 0.11 e Å^−^^3^
155 parameters	Δρ_min_ = −0.11 e Å^−^^3^
0 restraints	Extinction correction: *SHELXL2013*
0 constraints	Extinction coefficient: 0.0047 (18)
Primary atom site location: structure-invariant direct methods	

### Fractional atomic coordinates and isotropic or equivalent isotropic displacement parameters (Å^2^)

**Table:**

	*x*	*y*	*z*	*U*_iso_*/*U*_eq_	
C2	0.48635 (19)	0.3169 (3)	0.11626 (7)	0.0677 (5)	
H2	0.4743	0.4587	0.1328	0.081*	
C4	0.31624 (17)	0.4186 (3)	0.02499 (7)	0.0603 (4)	
O1	0.58960 (17)	−0.0451 (2)	0.13196 (6)	0.0977 (5)	
C3	0.41537 (18)	0.2726 (3)	0.06357 (7)	0.0657 (4)	
H3	0.4308	0.1283	0.0491	0.079*	
C1	0.58376 (19)	0.1497 (3)	0.14973 (7)	0.0680 (5)	
F1	0.02919 (14)	0.8135 (2)	−0.08612 (6)	0.1173 (5)	
C10	0.67648 (18)	0.2155 (3)	0.20579 (7)	0.0620 (4)	
C8	0.1728 (2)	0.7614 (3)	0.00570 (9)	0.0815 (5)	
H8	0.1411	0.9022	0.018	0.098*	
C5	0.2652 (2)	0.3477 (3)	−0.03154 (7)	0.0733 (5)	
H5	0.2962	0.2074	−0.0445	0.088*	
C9	0.2692 (2)	0.6298 (3)	0.04281 (8)	0.0735 (5)	
H9	0.3034	0.6827	0.0804	0.088*	
C7	0.1246 (2)	0.6831 (3)	−0.04919 (9)	0.0791 (5)	
C6	0.1692 (2)	0.4803 (3)	−0.06940 (8)	0.0818 (6)	
H6	0.1363	0.4317	−0.1076	0.098*	
C15	0.7800 (2)	0.0620 (3)	0.23197 (9)	0.0910 (6)	
H15	0.7907	−0.0775	0.214	0.109*	
C11	0.6644 (2)	0.4203 (3)	0.23317 (8)	0.0818 (5)	
H11	0.5962	0.5282	0.2162	0.098*	
C13	0.8525 (2)	0.3131 (4)	0.31101 (8)	0.0909 (6)	
H13	0.9105	0.3453	0.3466	0.109*	
C12	0.7521 (3)	0.4682 (4)	0.28558 (9)	0.0928 (6)	
H12	0.7425	0.6077	0.3037	0.111*	
C14	0.8678 (3)	0.1097 (4)	0.28394 (10)	0.1066 (8)	
H14	0.9376	0.0036	0.3007	0.128*	

### Atomic displacement parameters (Å^2^)

**Table:**

	*U*^11^	*U*^22^	*U*^33^	*U*^12^	*U*^13^	*U*^23^
C2	0.0750 (11)	0.0601 (10)	0.0658 (10)	0.0067 (8)	−0.0043 (8)	−0.0066 (8)
C4	0.0562 (9)	0.0621 (9)	0.0614 (9)	−0.0056 (8)	−0.0005 (7)	−0.0005 (8)
O1	0.1263 (12)	0.0693 (8)	0.0905 (9)	0.0259 (7)	−0.0263 (8)	−0.0201 (7)
C3	0.0668 (10)	0.0602 (9)	0.0688 (10)	0.0014 (8)	−0.0003 (8)	−0.0053 (8)
C1	0.0740 (11)	0.0621 (10)	0.0665 (10)	0.0091 (8)	−0.0008 (8)	−0.0073 (8)
F1	0.1054 (9)	0.1264 (10)	0.1130 (9)	0.0174 (7)	−0.0265 (7)	0.0378 (8)
C10	0.0658 (10)	0.0610 (9)	0.0587 (9)	0.0044 (8)	0.0028 (7)	−0.0012 (7)
C8	0.0795 (12)	0.0741 (12)	0.0888 (13)	0.0132 (10)	−0.0026 (10)	0.0048 (10)
C5	0.0779 (11)	0.0716 (11)	0.0683 (11)	−0.0064 (9)	−0.0043 (9)	−0.0043 (9)
C9	0.0758 (11)	0.0729 (11)	0.0699 (11)	0.0071 (9)	−0.0032 (9)	−0.0054 (9)
C7	0.0642 (11)	0.0878 (13)	0.0824 (13)	−0.0012 (10)	−0.0073 (9)	0.0216 (11)
C6	0.0807 (12)	0.0935 (14)	0.0675 (11)	−0.0128 (11)	−0.0132 (9)	0.0056 (10)
C15	0.1073 (15)	0.0710 (11)	0.0887 (13)	0.0170 (11)	−0.0233 (11)	−0.0078 (10)
C11	0.0900 (13)	0.0775 (12)	0.0751 (11)	0.0183 (10)	−0.0077 (10)	−0.0150 (9)
C13	0.1022 (15)	0.1003 (15)	0.0659 (11)	−0.0081 (13)	−0.0140 (10)	−0.0003 (11)
C12	0.1098 (16)	0.0867 (13)	0.0791 (13)	0.0040 (12)	−0.0051 (12)	−0.0251 (11)
C14	0.1241 (18)	0.0903 (14)	0.0954 (15)	0.0148 (13)	−0.0433 (13)	−0.0021 (12)

### Geometric parameters (Å, º)

**Table:**

C2—C3	1.317 (2)	C5—C6	1.383 (2)
C2—C1	1.467 (2)	C5—H5	0.93
C2—H2	0.93	C9—H9	0.93
C4—C5	1.380 (2)	C7—C6	1.356 (3)
C4—C9	1.388 (2)	C6—H6	0.93
C4—C3	1.453 (2)	C15—C14	1.370 (3)
O1—C1	1.2255 (19)	C15—H15	0.93
C3—H3	0.93	C11—C12	1.378 (2)
C1—C10	1.490 (2)	C11—H11	0.93
F1—C7	1.3615 (19)	C13—C12	1.358 (3)
C10—C11	1.372 (2)	C13—C14	1.365 (3)
C10—C15	1.374 (2)	C13—H13	0.93
C8—C7	1.356 (3)	C12—H12	0.93
C8—C9	1.372 (2)	C14—H14	0.93
C8—H8	0.93		
			
C3—C2—C1	122.11 (15)	C4—C9—H9	119.5
C3—C2—H2	118.9	C8—C7—C6	122.65 (17)
C1—C2—H2	118.9	C8—C7—F1	119.12 (19)
C5—C4—C9	117.80 (15)	C6—C7—F1	118.23 (18)
C5—C4—C3	119.76 (15)	C7—C6—C5	118.00 (17)
C9—C4—C3	122.44 (15)	C7—C6—H6	121
C2—C3—C4	128.77 (15)	C5—C6—H6	121
C2—C3—H3	115.6	C14—C15—C10	121.52 (18)
C4—C3—H3	115.6	C14—C15—H15	119.2
O1—C1—C2	120.43 (15)	C10—C15—H15	119.2
O1—C1—C10	119.37 (15)	C10—C11—C12	120.78 (17)
C2—C1—C10	120.20 (14)	C10—C11—H11	119.6
C11—C10—C15	117.77 (16)	C12—C11—H11	119.6
C11—C10—C1	123.94 (15)	C12—C13—C14	119.57 (18)
C15—C10—C1	118.29 (15)	C12—C13—H13	120.2
C7—C8—C9	118.99 (18)	C14—C13—H13	120.2
C7—C8—H8	120.5	C13—C12—C11	120.46 (18)
C9—C8—H8	120.5	C13—C12—H12	119.8
C4—C5—C6	121.60 (17)	C11—C12—H12	119.8
C4—C5—H5	119.2	C13—C14—C15	119.88 (19)
C6—C5—H5	119.2	C13—C14—H14	120.1
C8—C9—C4	120.94 (17)	C15—C14—H14	120.1
C8—C9—H9	119.5		
			
C1—C2—C3—C4	−179.76 (16)	C9—C8—C7—C6	−0.8 (3)
C5—C4—C3—C2	173.35 (17)	C9—C8—C7—F1	−179.91 (16)
C9—C4—C3—C2	−6.9 (3)	C8—C7—C6—C5	1.4 (3)
C3—C2—C1—O1	−7.3 (3)	F1—C7—C6—C5	−179.52 (16)
C3—C2—C1—C10	172.77 (16)	C4—C5—C6—C7	−0.7 (3)
O1—C1—C10—C11	−171.95 (19)	C11—C10—C15—C14	0.5 (3)
C2—C1—C10—C11	8.0 (3)	C1—C10—C15—C14	−179.2 (2)
O1—C1—C10—C15	7.7 (3)	C15—C10—C11—C12	−0.8 (3)
C2—C1—C10—C15	−172.33 (18)	C1—C10—C11—C12	178.88 (17)
C9—C4—C5—C6	−0.6 (2)	C14—C13—C12—C11	0.8 (3)
C3—C4—C5—C6	179.19 (16)	C10—C11—C12—C13	0.1 (3)
C7—C8—C9—C4	−0.5 (3)	C12—C13—C14—C15	−1.1 (4)
C5—C4—C9—C8	1.2 (3)	C10—C15—C14—C13	0.4 (4)
C3—C4—C9—C8	−178.60 (16)		

### Hydrogen-bond geometry (Å, º)

**Table:**

*D*—H···*A*	*D*—H	H···*A*	*D*···*A*	*D*—H···*A*
C5—H5···O1^i^	0.93	2.49	3.244 (2)	138
C13—H13···F1^ii^	0.93	2.68	3.465 (2)	142

Symmetry codes: (i) −*x*+1, −*y*, −*z*; (ii) *x*+1, −*y*+3/2, *z*+1/2.
